# Aramchol downregulates stearoyl CoA-desaturase 1 in hepatic stellate cells to attenuate cellular fibrogenesis

**DOI:** 10.1016/j.jhepr.2021.100237

**Published:** 2021-01-28

**Authors:** Dipankar Bhattacharya, Brittany Basta, Jose M. Mato, Amanda Craig, David Fernández-Ramos, Fernando Lopitz-Otsoa, Darya Tsvirkun, Liat Hayardeny, Vasuretha Chandar, Robert E. Schwartz, Augusto Villanueva, Scott L. Friedman

**Affiliations:** 1 Division of Liver Diseases, Liver Cancer Program, Tisch Cancer Institute, Icahn School of Medicine at Mount Sinai, New York, NY, USA; 2 Precision Medicine and Metabolism Laboratory, CIC BioGUNE BRTA, Centro de Investigación Biomédica en Red de Enfermedades Hepáticas y Digestivas (CIBERehd), 48160, Derio, Bizkaia, Spain; 3 Galmed Pharmaceuticals, Tel Aviv, Israel; 4 Department of Medicine, Weill Cornell Medical, New York, NY, USA; 5 Department of Physiology, Biophysics, and Systems Biology, Weill Cornell Medical, New York, NY, USA

**Keywords:** Fibrosis, Non-alcoholic steatohepatitis, Fatty liver disease, Hepatic fibrosis, ABCA1, ATP-binding cassette transporter 1, EMT, epithelial-mesenchymal-transition, GSEA, gene set enrichment analysis, GSH, glutathione, GSSG, glutathione disulfide, Hh, Hedgehog, HRP, horse radish peroxidase, HSC, hepatic stellate cell, MCD, methionine-choline depleted diet, MMP-2, matrix metalloproteinase 2, MUFAs, monounsaturated fatty acids, NAFLD, non-alcoholic fatty liver disease, NASH, non-alcoholic steatohepatitis, PDMS, polydimethylsiloxane, phHeps, primary human hepatocytes, phHSCs, primary human hepatic stellate cells, PPAR, peroxisome proliferator-activated receptor, SCD1, stearoyl CoA-desaturase 1, siRNA, small inhibitory RNA, SMA, smooth muscle actin, TAA, thioacetamide

## Abstract

**Background & Aims:**

Aramchol is a fatty acid-bile acid conjugate that reduces liver fat content and is being evaluated in a phase III clinical trial for non-alcoholic steatohepatitis (NASH). Aramchol attenuates NASH in mouse models and decreases steatosis by downregulating the fatty acid synthetic enzyme stearoyl CoA desaturase 1 (SCD1) in hepatocytes. Although hepatic stellate cells (HSCs) also store lipids as retinyl esters, the impact of Aramchol in this cell type is unknown.

**Methods:**

We investigated the effects of Aramchol on a human HSC line (LX-2), primary human HSCs (phHSCs), and primary human hepatocytes (phHeps).

**Results:**

In LX-2 and phHSCs, 10 μM Aramchol significantly reduced *SCD1* mRNA while inducing *PPARG* (*PPARγ*) mRNA, with parallel changes in the 2 proteins; *ACTA2*, *COL1A1*, *β-PDGFR* (*bPDGFR*) mRNAs were also significantly reduced in LX-2. Secretion of collagen 1 (Col1α1) was inhibited by 10 μM Aramchol. *SCD1* knockdown in LX-2 cells phenocopied the effect of Aramchol by reducing fibrogenesis, and addition of Aramchol to these cells did not rescue fibrogenic gene expression. Conversely, in LX-2 overexpressing SCD1, Aramchol no longer suppressed fibrogenic gene expression. The drug also induced genes in LX-2 that promote cholesterol efflux and inhibited *ACAT2*, which catalyses cholesterol synthesis. In phHeps, Aramchol also reduced *SCD1* and increased *PPARG* mRNA expression.

**Conclusions:**

Aramchol downregulates *SCD1* and elevates *PPARG* in HSCs, reducing *COL1A1* and *ACTA2* mRNAs and COL1A1 secretion. These data suggest a direct inhibitory effect of Aramchol in HSCs through SCD1 inhibition, as part of a broader impact on both fibrogenic genes as well as mediators of cholesterol homeostasis. These findings illustrate novel mechanisms of Aramchol activity, including potential antifibrotic activity in patients with NASH and fibrosis.

**Lay summary:**

In this study, we have explored the potential activity of Aramchol, a drug currently in clinical trials for fatty liver disease, in blocking fibrosis, or scarring, by hepatic stellate cells, the principal collagen-producing (*i.e.* fibrogenic) cell type in liver injury. In both isolated human hepatic stellate cells and in a human hepatic stellate cell line, the drug suppresses the key fat-producing enzyme, stearoyl CoA desaturase 1 (SCD1), which leads to reduced expression of genes and proteins associated with hepatic fibrosis, while inducing the protective gene, PPARγ. The drug loses activity when SCD1 is already reduced by gene knockdown, reinforcing the idea that inhibition of SCD1 is a main mode of activity for Aramchol. These findings strengthen the rationale for testing Aramchol in patients with NASH.

## Highlights

• The antifibrotic activity of Aramchol was assessed in human hepatic stellate cells (HSCs).
• Aramchol reduces fibrogenic gene expression by inhibiting SCD1 and inducing PPARγ.
• Aramchol inhibits pathways that increase HSC cholesterol content.
• The antifibrotic activity of Aramchol reinforces its potential efficacy in human NASH.

## Graphical abstract

**Figure:**
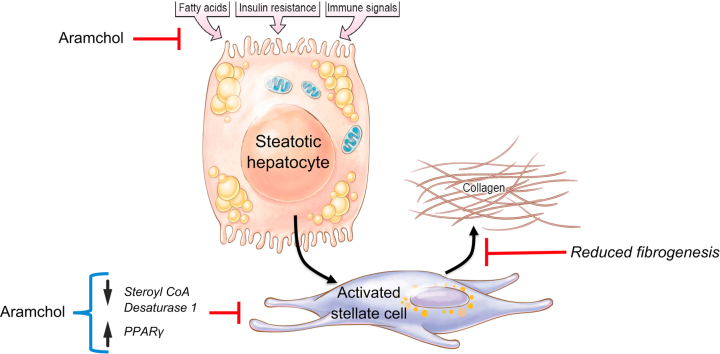

## Introduction

Liver fibrosis and its most advanced stage, cirrhosis, is the 11th most common cause of death worldwide.^1^ Several chronic diseases promote hepatic fibrosis, including viral hepatitis, immune injury, and alcoholic and non-alcoholic steatohepatitis (NASH). NASH is the advanced form of non-alcoholic fatty liver disease (NAFLD), deﬁned histologically by the presence of hepatic fat (steatosis) with inflammation and hepatocellular ballooning. Progression of NASH results from multiple parallel events that converge upon hepatocytes, including steatosis, oxidative stress, mitochondrial dysfunction, apoptosis, and inflammation, which collectively provoke fibrosis.^2^^,^^3^

In normal liver, hepatic stellate cells (HSCs) are a resident perisinusoidal mesenchymal cell type that contributes to liver homeostasis, but during injury they transdifferentiate from a quiescent to an activated state, with loss of vitamin A-containing lipid droplets and increased production of collagen and other extracellular matrix constituents.^3^ The mechanistic link between loss of lipids in HSCs and the development of hepatic fibrosis is not well understood, but is thought to involve consumption of fatty acids by increased autophagy.^4^

Activation of HSCs drives hepatic fibrosis, and is inhibited by peroxisome proliferator-activated receptor gamma (PPARγ) signalling, in part through antagonism of Wnt activity.^5^ PPARγ agonists have been investigated as a treatment for liver fibrosis by inhibiting collagen production by activated HSCs, but enthusiasm has been limited by concern about weight gain induced by these agents.

Among therapies for NASH currently in clinical development,^2^ arachidyl amido cholanoic acid (Aramchol) is a fatty acid-bile acid conjugate that attenuates experimental fibrosis in 2 animal models of NASH,^6^ as well as in a chemical-induced fibrosis model caused by thioacetamide.^7^ Aramchol targets stearoyl-CoA desaturase 1 (SCD1),^8^ the rate-limiting step in the synthesis of monounsaturated fatty acids (MUFAs), the major fatty acid of triglycerides, cholesteryl esters, and membrane phospholipids,^9^^,^^10^ through an unknown mechanism. Aramchol also increases metabolite flux through the trans-sulfuration pathway, leading to a rise in glutathione (GSH) and the GSH/glutathione disulfide (GSSG) ratio, which preserve cellular antioxidant levels and intracellular redox status. SCD1 deficiency in mice reduces lipid synthesis and increases mitochondrial fatty acid β-oxidation and insulin sensitivity in several tissues including the liver and prevents hepatic steatosis in mouse models of NASH^9^^,^^11^; these data point to SCD1 as a potential therapeutic target in NASH.

In a phase IIb trial in humans, Aramchol improved NASH resolution and fibrosis in a larger proportion of participants compared with placebo, in a dose-dependent manner.^12^ Aramchol is currently being evaluated in a phase III clinical trial for patients with NASH (ClinicalTrials.gov Identifier: NCT04104321).

Whereas Aramchol reduces liver fat by downregulating SCD1 in hepatocytes, the activity of Aramchol towards HSCs and specifically its effect on fibrogenesis are less clear. In this study, we have assessed the impact of Aramchol on fibrogenesis by HSCs. Aramchol downregulates SCD1 in LX-2 cells, a well-validated human HSC line,^13^ in primary human hepatic stellate cells (phHSCs), and in primary human hepatocytes (phHeps), and direct SCD1 depletion using siRNA phenocopies the inhibitory effects of Aramchol on HSC fibrogenesis.

## Materials and methods

### Aramchol formulation

The investigational agent Aramchol (arachidyl amido cholanoic acid) (F.W. 702.118) was developed by Galmed Pharmaceuticals (Tel Aviv, Israel).^14^ The purity of Aramchol is >99% by HPLC and was initially reconstituted at 10 mM concentration in DMSO as stock solution before each experiment. A serial dilution to 5 and 10 μM working concentration of Aramchol was prepared from 10 mM freshly made stock solution in serum-free media (‘starvation media’) and incubated with HSCs or hepatocytes. The final concentration of 10 μM was chosen because this corresponds to the predicted therapeutic Cmax of the drug *in vivo* based on pharmacokinetic studies in rodents.

### LX-2 cell culture

LX-2 cells, an immortalised human HSC line,^13^ were serum-starved overnight to quiesce and synchronise metabolic activity in serum-free DMEM (Thermo Fisher Scientific, MA, USA) supplemented with 0.1% BSA, without antibiotics at 37°C. All experiments using LX-2 cells were repeated 3 times, each in triplicate.

### phHSCs

phHSCs were isolated from anonymous, Institutional Review Board (IRB)-exempt, surgically resected human livers^15^ from 3 patient donors (Patients 1, 2, and 3), as approved by the IRB at the Icahn School of Medicine at Mount Sinai, NY, USA. Relevant information about the patient donors is listed in Table S1. After passaging twice with trypsin, the culture-activated phHSCs were stored in liquid nitrogen until further use. Purity of the phHSCs was consistently >93% as determined by immunostaining with antibodies to desmin, glial fibrillary acidic protein (GFAP), alpha smooth muscle actin (αSMA), as well as CD31 (for liver sinusoidal endothelial cells) and CD68 (for hepatic macrophages). All experiments with phHSCs were done in triplicate followed by 3 biological repeats in each individual patient sample.

### phHeps

phHeps were obtained from BioIVT (New York, NY, USA), which maintain robust differentiated function. To culture these cells, polydimethylsiloxane (PDMS) microwells were autoclaved, placed in a 12-well plate and coated with 1% (w/v) Pluronic® F127 (Sigma) solution before cell seeding to prevent cell attachment on the PDMS surface and facilitate formation of cell aggregates. To form cell aggregates, cell suspensions of phHep (120,000 cells) were added to each well of a 24-well plate with PDMS microwells inside. The cells that fell into the microwells formed cell aggregates after overnight culture with gentle shaking. The aggregates were cultured in microwells for 8 days before incubation with Aramchol. Purity and authentication of phHeps were assessed by measurement of secreted albumin and α1-Antitrypsin (AAT) by ELISA. CYP3A4 activity assay of the cells was evaluated using P450-Glo assay (Promega, WI, USA). Each of 3 experiments using phHep cells was performed in triplicate.

### Cell viability assay

LX-2 cells (5,000/well) or phHSCs (10,000/well) were plated in 96-well plates. After serum-starvation overnight the cells were incubated with incremental concentrations of Aramchol in serum-free media for 24 or 48 h. Cell viability assays were performed using CellTiter 96 Aqueous One Solution Cell Proliferation Assay kit (Promega) according to the manufacturer’s protocol, and the absorbance was measured at 490 nm.

### Cell proliferation assay

LX-2 cells (5,000/well) or phHSCs (10,000/well) were plated in 96-well plates. After serum starvation overnight the cells were incubated at the indicated concentrations of Aramchol in serum-free media for 24 and 48 h. Following drug incubation, the cells were labelled with BrdU for either 2 h (for LX-2 cells) or 16 h (for phHSCs) at 37°C. Cell proliferation was quantified using the cell proliferation ELISA, BrdU colorimetric kit (Roche, NY, USA) according to the manufacturer’s instructions. Absorbance was measured at 370 nm with reference wavelength at 492 nm.

### Real time PCR assessment of gene expression

LX-2 cells (150,000/well) or phHSCs (200,000/well) were seeded in 6-well plates. Cells were incubated overnight in starvation media, then 5 or 10 μM Aramchol or DMSO vehicle was added in starvation media for 24 or 48 h. For cultured phHeps, the cells were incubated with 5 or 10 μM Aramchol or DMSO for 24 or 48 h. Cells were harvested and total RNA was extracted using an RNeasy Mini Kit (Qiagen, CA, USA). A 500-ng sample of total RNA was used for reverse transcription with an RNA to cDNA EcoDry Premix (Double Primed) Kit (Clontech, CA, USA). Expression of fibrogenic genes was quantified using RT-qPCR with iQ SYBR Green Supermix (Bio-Rad, CA, USA) on a LightCycler 480 II (Roche Diagnostics Corporation, IN, USA) instrument. *GAPDH* was used as a housekeeping gene control to normalise expression. The primer sequences used for RT-qPCR are shown in Table S2. Total mRNA purification, quantification, and purity test (A_260_/A_280_), cDNA synthesis (reverse transcript), qPCR primer synthesis and reaction, qPCR validation (including selection of a housekeeping gene that was uniformly expressed across all treatment groups), target gene expression data analysis (fold change), documentations, and quality control were performed strictly according to the Minimum Information for Publication of Quantitative Real-Time PCR Experiment guidelines.

### Protein quantification

LX-2 cells (150,000 cells/well) or phHSC (200,000/well) were seeded in 6-well plates. Cells were incubated overnight in serum-free media, then with 5 or 10 μM Aramchol or DMSO vehicle for the durations indicated. Cells were harvested and lysed using RIPA buffer along with Pierce Protease Inhibitor Mini Tablets, EDTA-Free (Thermo Scientific, IL, USA). Total protein was measured by Bradford colorimetric assay (Bio-Rad). A 10-μg sample of total protein was loaded and separated by NuPAGE 4–12% Bis-Tris gels (Thermo Scientific). After transfer to a polyvinylidene difluoride (PVDF) membrane the blot was blocked by 5% non-fat dry milk (Thermo Scientific) in 1× PBS. Primary antibodies were rabbit anti-PPARG (Abcam, MA, USA), rabbit anti-αSMA (Abcam), mouse anti-SCD1 (Abcam) and mouse anti-GAPDH (Millipore, CA USA). Horse radish peroxidase (HRP)-conjugated secondary antibodies were either goat anti-rabbit IgG-HRP (Jackson ImmunoResearch, PA, USA) or anti-mouse IgG-HRP (Cell Signaling Technology, MA, USA). The membrane was treated with Immobilon Western Chemiluminescent HRP substrate (Millipore) and the signal was captured using an Amersham Imager 6000 (GE Healthcare, PA, USA). Protein bands of 54 kDa (PPARG), 42 kDa (αSMA), 32 kDa (SCD1), and 37 kDa (GAPDH) were quantified by densitometry, followed by exporting of images and analysis with ImageJ 1.50f software (http://imagej.nih.gov/ij) and relative expression normalised to a GAPDH loading control.

### ELISA for secreted Col1α1 protein

Secreted Col1α1 was quantified in cell culture media from Aramchol-treated LX-2 or phHSCs. For the 48h time point, fresh medium was added at 24 h and incubated for an additional 24 h before collection. Then, the media were centrifuged at 1,200 rpm for 10 min to remove cellular debris. The sample was diluted 1:1,000 into diluent buffer, and secreted Col1α1 measured using a Human Pro-Collagen I alpha 1 SimpleStep ELISA kit (Abcam) with human Pro-Collagen1α1 as a standard, according to the manufacturer’s protocol, with absorbance was measured at 450 nm.

### Total RNA isolation, mRNA expression profiling

LX-2 cells (150,000/well) were seeded in 6-well plates. After overnight culture in starvation media cells were incubated with either 10 μM Aramchol or DMSO vehicle for the indicated durations. Cells were harvested and total RNA was extracted using RNeasy Mini Kit as described above. RNA Integrity Number was assessed using a 2100 Bioanalyzer. Sequencing libraries were made with the TruSeq Stranded mRNA Library Prep Kit according to the manufacturer’s suggestions. Sequencing parameters are as follows: poly-A RNA enrichment, 175-bp paired reads ran on a HiSeq2500 Illumina instrument. Mapping of raw sequencing reads was performed using STAR (Spliced Transcript Alignments to a Reference; 2.4.0c) to the UCSC GRCh37/hg19 reference genome.^16^ RSeQC and Picard was used to assess quality control based on concordance using principal component analysis (Fig. S1).^17^

### RNA-seq data analysis

Differential gene expression was calculated using the DESeq2 package in R.^18^ Gene set enrichment analysis (GSEA) was used to find enrichment of Hallmark, REACTOME, and KEGG gene sets between the 3 different treatment conditions (FDR <0.05).^19^^,^^20^ A running sum statistic was used to determine enrichment scores at the maximum deviation from zero.

### RNA interference-mediated SCD1 knockdown

To knock down SCD1 in HSCs, we used a human SCD1 small interfering RNA (SMART pool: Accell SCD siRNA; Accession no.: NM_005063), or control Accell red non-targeting siRNA (red fluorescent tagged negative control), 5× siRNA buffer (300 mM KCl, 30 mM HEPES [pH 7.5] 1.0 mM MGCl_2_) and Accell siRNA delivery media (Horizon Discovery, CO, USA). siRNA was reconstituted in 1× siRNA buffer (made from 5× siRNA in RNase-free water) according to the manufacturer’s instructions. LX-2 cells (70,000/well) were seeded in 12-well culture plates, and when at 60–70% confluency the cells were transfected with either 0.5 μM SCD1 siRNA, 0.5 μM red fluorescent non-targeting siRNA, or 1× siRNA buffer (referred as untransfected) in the Accell siRNA delivery media (without antibiotics). Fresh media were added each 24 h, and incubation continued as indicated. At 48-, 72-, and 96-h post-transfection the cells were harvested, total mRNA was purified and RT-qPCR of fibrogenic genes was performed, as described above (using *GAPDH* as a control housekeeping gene). After siRNA-mediated knockdown of SCD1 in LX-2 cells, both cells and culture media were collected, and αSMA and SCD1 protein expression was quantified by densitometry of Western blot and normalised to expression of GAPDH, as described above. Secreted Col1α1 in media from LX-2 cells following siRNA knockdown was measured with ELISA, as described above. To assess the efficiency of siRNA transfection, LX-2 cells transfected with red fluorescent non-targeting siRNA were washed in 1× PBS and imaged. Briefly, the fluorescence signal from live cells was generated using X-Cite 120LED Boost powerpack (Excelitas Technologies, MA, USA) by excitation at 550 nm and the emission signal at 570 nm was captured with an Axiocam 105 colour digital camera (Zeiss, NY, USA) using a 10×/0.45 numerical aperture objective in an Axio Observer 7 inverted microscope (Zeiss). Images in the same focal plane were captured in the phase contrast channel as well (Fig. S2). To assess the effect of Aramchol in LX-2 with SCD1 siRNA knockdown, 24 h after knockdown either DMSO (as vehicle) or 10 μM Aramchol was added in the culture media and the knockdown cells were maintained for an additional 48 h (72 h of siRNA post-transfection), after which total mRNA was purified and mRNAs were quantified by RT-qPCR as described above.

### Aramchol treatment of HSCs with SCD1 overexpression

C-terminal Myc-DDK Flag tagged pCMV6-Entry empty vector (CAT# PS100001) and 1077 bp human SCD1 encoded cloned gene (accession# NM_005063) in the same expression vector (CAT# RC209148) were commercially purchased from Origene (MD, USA) and overexpressed in LX-2 cells. After seeding 150,000/well in 6-well plates, transient transfection was performed of empty vector or SCD1 expression vector using Lipofectamine 2000 (Thermo Fisher Scientific) transfection reagent according to manufacturer’s protocol. Briefly, after making the transfection complex with Lipofectamine 2000 and 2 μg of respective plasmid DNAs, transfection in LX-2 cells was carried out in presence of OPTI-MEM (Thermo Fisher Scientific) overnight at 37°C. The next day, the transfection media were replaced with serum-free media, with or without 10 μM Aramchol. The cells were harvested 48 h after adding the drug, total mRNA was purified and gene expression was assessed using RT-qPCR.

### Graphs and statistical analyses

Graphs and statistical analyses were performed using Prism 6 (GraphPad). Experiment data are reported as mean ± SEM and was subjected to a 2-tailed Student *t* test with a 95% confidence interval. Values of *p* <0.05 were considered significant.

## Results

### Aramchol downregulates fibrogenic gene expression in HSCs

To select the optimal efficacy of Aramchol we first assessed cell viability and proliferation in LX-2 and phHSCs using incremental drug concentrations up to 20 μM. Both cell types tolerated up to 10 μM Aramchol for 48 h, with no impact on cell viability or proliferation (Fig. S3). LX-2 cells were incubated with 5 or 10 μM of the drug for 24 or 48 h, followed by quantification of mRNAs that reflect HSC activation, including alpha-smooth muscle actin (*ACTA2*), collagen type I alpha-1 chain (*COL1A1*), beta platelet derived growth factor (*bPDGFR*), and matrix metalloproteinease 2 (*MMP2*), in addition to *PPARG* and *SCD1* mRNAs.

**Fig. 1:**
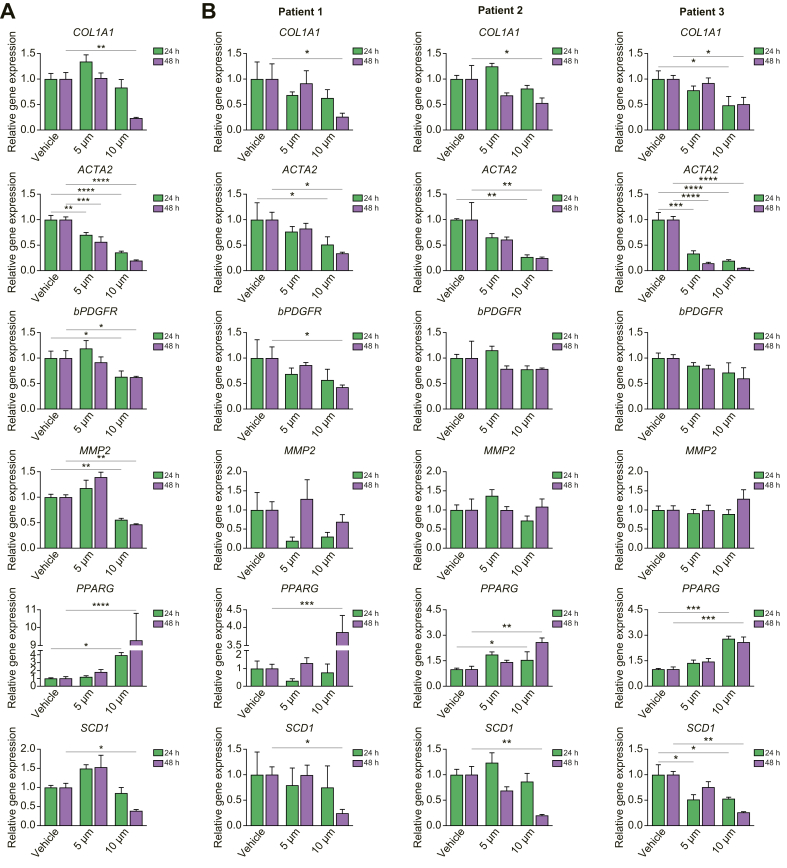
Aramchol significantly downregulates *SCD1* mRNA and fibrogenic genes, and upregulates *PPARG* mRNA in HSCs. (A) LX-2 cells were treated with 5 μM and 10 μM Aramchol or DMSO (vehicle) for 24 or 48 h. Gene expression of *SCD1*, *PPARG*, and fibrotic markers were evaluated by RT-qPCR. Dose-dependent inactivation of *COL1A1*, *ACTA2*, *bPDGFR*, and *MMP2* along with *SCD1* and simultaneous activation of *PPARG* mRNA are shown. (B) Primary human hepatic stellate cells isolated from 3 donors (Patients 1, 2, and 3) were treated with Aramchol as described in panel A for LX-2 cells. Dose-dependent downregulation of *COL1A1* and *ACTA2* along with *SCD1* expression and activation of *PPARG* are shown. Data were normalised to *GAPDH* used as internal control. Results are reported as means ± SEM (n = 3). ∗*p* <0.05, ∗∗*p* <0.01, ∗∗∗*p* <0.001, ∗∗∗∗*p* <0.0001 using 2-tailed Studentàs *t* test with a 95% confidence interval. HSCs, hepatic stellate cells; PPARG, peroxisome proliferator-activated receptor gamma; SCD1, stearoyl CoA-desaturase 1.

At 24 h of incubation, 10 μM Aramchol reduced the expression *ACTA2, bPDGFR*, and *MMP2* mRNAs while it induced *PPARG*, which was sustained at 48 h (Fig. 1A); there was also reduction of *ACTA2* and *MMP2* at 48 h*,* along with *COL1A1*and *SCD1* mRNAs (Fig. 1A). The effect of Aramchol (10 μM) was replicated in each of 3 patient-derived primary HSC isolates treated for up to 48 h, with similar downregulation in fibrogenic genes and *SCD1* mRNA (Fig. 1B). Aramchol also induced *PPARG* mRNA at 48 h in phHSCs (Fig. 1B).

**Fig. 2:**
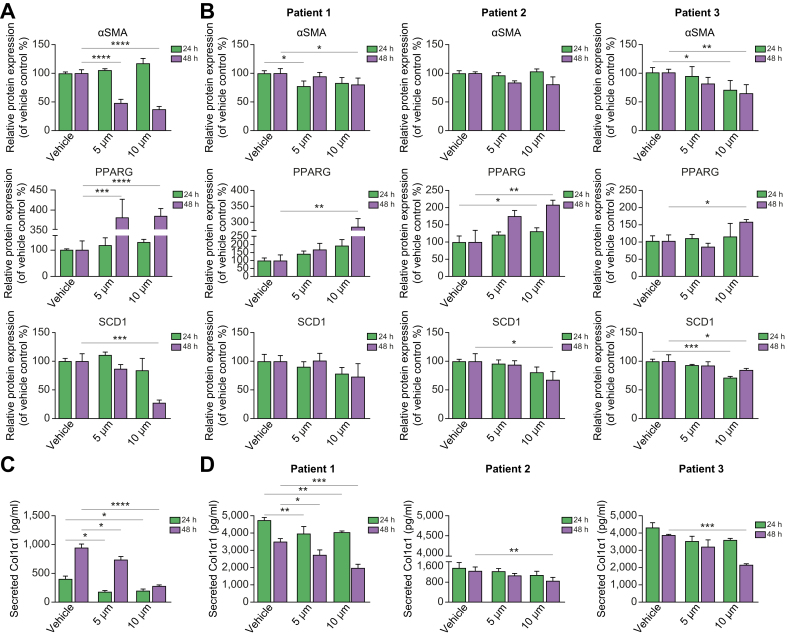
Aramchol inhibits Col1α1, αSMA, SCD1 and upregulates PPARG proteins in HSCs. (A) LX-2 cells were treated with 5 μM and 10 μM Aramchol or DMSO (vehicle) for 24 or 48 h. Protein expression was quantified by densitometry of αSMA, PPARG, and SCD1 bands in Western blots in Fig. S4A. Dose-dependent downregulation of αSMA and SCD1 and upregulation of PPARG were assessed. (B) Primary human hepatic stellate cells isolated from 3 donor livers were treated with Aramchol as described in panel A for LX-2 cells. Expression of αSMA, PPARG, and SCD1 proteins was analysed by densitometry of Western blots shown in Fig. S4B. Downregulation of αSMA, SCD1 and upregulation of PPARG protein expression by Aramchol treatment were evident. Data were normalised to GAPDH. (C) Secreted Col1α1 in culture media (both vehicle and Aramchol treated) from LX-2 cells was measured by ELISA using human pro-collagen 1a1 as a standard. Dose-dependent reduction of Col1α1 in culture media is evident. (D) Soluble Col1α1 protein in culture media was reduced by Aramchol in 3 isolates of phHSCs. Results are reported as means ± SEM (n = 3). ∗*p* <0.05, ∗∗*p* <0.01, ∗∗∗*p* <0.001, ∗∗∗∗*p* <0.0001 using 2-tailed Student's *t* test with a 95% confidence interval. HSCs, hepatic stellate cells; PPARG, peroxisome proliferator-activated receptor gamma; SCD1, stearoyl CoA-desaturase 1.

**Fig. 3:**
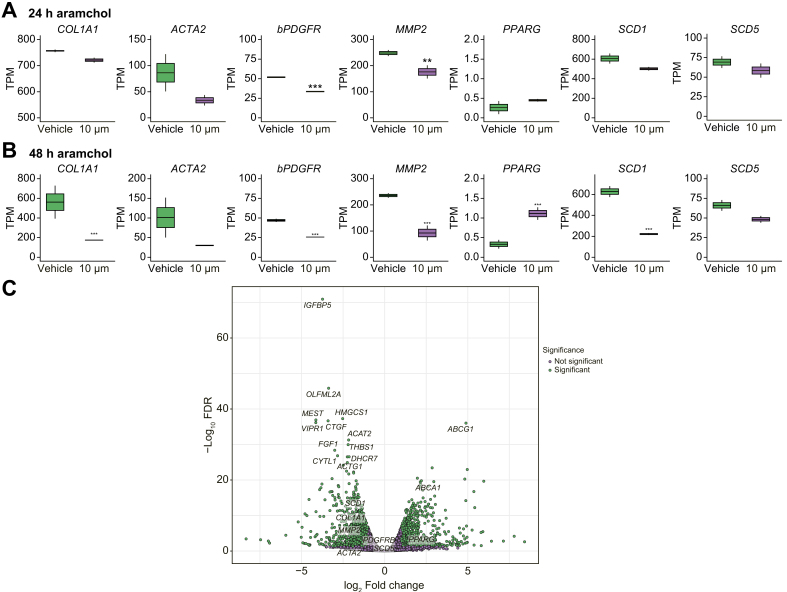
Global effects of Aramchol on HSC gene expression by RNA-seq. RNA was extracted from LX-2 cells treated with 10 μM Aramchol or DMSO (vehicle) for 24 (A) or 48 (B) h, followed by RNAseq. Fibrogenic genes as well as *SCD1* mRNA expression were significantly downregulated by Aramchol treatment at 48 h, consistent with findings from RT-qPCR (Fig. 1). In contrast, *SCD5* mRNA expression was not significantly affected by Aramchol. *PPARG* mRNA expression was upregulated compared with vehicle-treated cells. Results are reported as transcripts per million (TPM), n = 2. ∗*p* <0.05, ∗∗*p* <0.01, ∗∗∗*p* <0.001. The *p* values were calculated using DeSeq2 differential gene expression analysis and adjusted for multiple testing. (C) After 48 h of 10 μM Aramchol treatment in LX-2 cells the cholesterol efflux regulatory genes *ABCA1* and *ABCG1* were upregulated along with *PPARG. SCD1* was downregulated (but not *SCD5*) along with other fibrogenesis-related genes including *IGFBP5*, *OLFML2A*, and *CTFG*. Volcano plot of Log2 fold change plotted against -Log10 adjusted *p* value. Each dot represents 1 gene. Differentially expressed genes with a -Log10 adjusted *p* value greater than 1.3 and a Log2 fold change greater than 1 or less than -1 are highlighted in purple. All other genes are represented as grey. HSC, hepatic stellate cell; PPARG, peroxisome proliferator-activated receptor gamma; SCD1, stearoyl CoA-desaturase 1.

Expression of αSMA and SCD1 proteins was also inhibited by Aramchol in both LX-2 cells (Fig. 2A and S4A) and in 3 separate isolates of phHSCs (Fig. 2B and S4B). The gene expression changes measured by RT-qPCR were confirmed in the RNA-seq datasets at 24 and 48 h of treatment with 10 μM Aramchol (Fig. 3A and B) from LX-2 cells. The RNAseq data also demonstrate that unlike *SCD1*, the expression of *SCD5* mRNA, although reduced, was not significantly downregulated by Aramchol treatment (Fig. 3A–C).

### Aramchol reduces Col1α1 secretion in LX-2 cells and phHSCs

Activated HSCs constitutively secrete COL1A1 protein. To investigate the effect of Aramchol on Col1α1 secretion, culture media from both LX-2 and phHSCs were analysed using ELISA. In LX-2 cells there was a dose-dependent reduction by 10 μM Aramchol of 50% at 24 h, and 70% at 48 h, compared with vehicle-treated cells (Fig. 2C). The 3 isolates of phHSCs exhibited similar dose-dependent reductions by 10 μM Aramchol of 32%, 43%, and 44% in Col1α1 secretion at 48 h (Fig. 2D).

### Aramchol reduces cholesterol efflux genes, *ABCA1* and *ABCG1*, in HSCs

Analysis of the RNA-seq data uncovered a marked increase by Aramchol in gene expression for ATP-binding cassette transporter 1 (*ABCA1*) as depicted in a volcano plot (Fig. 3C). The product of this gene is a cholesterol efflux regulatory protein that acts as a major regulator of cholesterol homeostasis. Aramchol is known to directly stimulate *ABCA1* activity without affecting mRNA levels,^8^ but this is the first evidence of the induction of *ABCA1* mRNA by Aramachol. Similarly, Aramchol induced *ABCG1*, which also mediates cholesterol and phosopholid efflux,^21^ and downregulated *ACAT2*, which catalyses the synthesis of cholesterol esters.^22^ These changes would further reduce intracellular cholesterol in HSCs. Importantly, increased cholesterol accumulation in HSCs accelerates liver fibrosis, and thus enhanced efflux through *ABCA1* and *ABCG1* induction while reducing *ACAT2* may be additional mechanisms by which Aramchol reduces HSC fibrogenesis.^23^

**Fig. 4:**
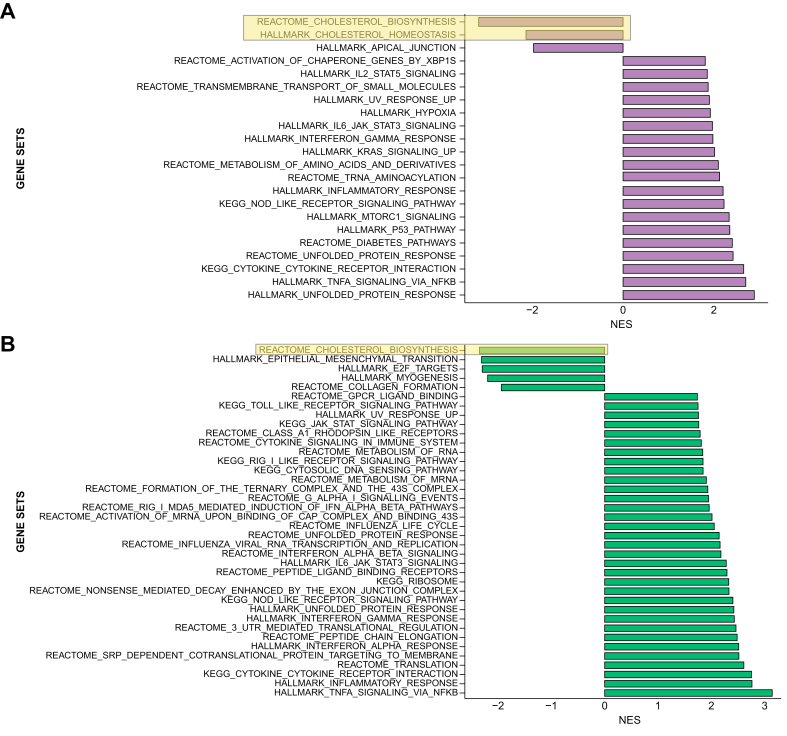
Aramchol downregulates epithelial-mesenchymal-transition (EMT) associated proteins, cholesterol biosynthesis pathways, and proteins associated with collagen production in HSCs. Gene set enrichment analysis of LX-2 cells after 24 (A) or 48 (B) h of Aramchol is shown. The x-axis represents normalised enrichment scores of gene sets. HSCs, hepatic stellate cells.

We performed GSEA to identify other cellular pathways responsive to Aramchol (Fig. 4A and B). Gene sets for both cholesterol biosynthesis and homeostasis were downregulated after 24 h of 10 μM Aramchol, and the downregulation of cholesterol biosynthesis persisted after 48 h of treatment. A full list of differentially expressed genes is available in Table S3. These findings support the possibility that the effect of Aramchol on cholesterol levels in HSCs may contribute to its antifibrotic activity.

### Aramchol attenuates the HSC activation phenotype

By GSEA, genes involved in collagen formation were downregulated after 48 h of treatment, indicating reduced HSC activation and fibrogenesis (Fig. 4) These findings are further supported by downregulation of fibrosis-associated genes including *IGFBP5*,^24^ *OLFML2A*,^25^ and *CTGF* (Fig. 3C), reinforcing the conclusion that Aramchol elicits a coordinated genetic program that broadly reduces fibrogenic activity by HSCs. Interestingly, 48 h of Aramchol reduced the expression of a gene set involved in epithelial-mesenchymal-transition (EMT; Fig. 4), a process in which epithelial cells acquire the phenotypic characteristics of mesenchymal cells, including increased motility and invasiveness. Quiescent liver cells express high levels of *PPARG* and low levels of Hedgehog (Hh) signalling components, while activated HSCs have increased Hh signalling that represses *PPARG* expression.^26^ The reduced expression of the EMT gene set in LX-2 cells by Aramchol could be another indirect mechanism underlying the ability of Aramchol to reduced activation of HSCs by upregulating *PPARG* to promote a more quiescent phenotype.

### RNAi-mediated *SCD1* knockdown reduces profibrotic gene and protein expression, and induces *PPARG*

**Fig. 5:**
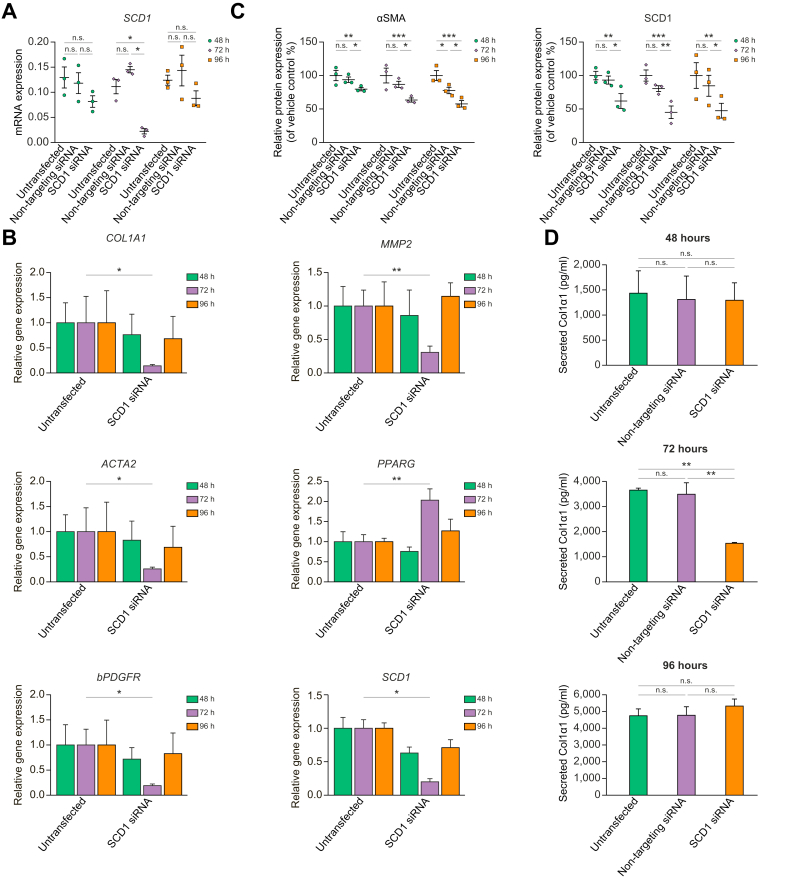
SCD1 knockdown reduces HSC activation and upregulates PPARG. LX2 cells were transfected with either a *SCD1* siRNA or non-targeting fluorescent red siRNA. At 48, 72, and 96 h after transfection the cells and culture media were harvested and total mRNA and protein were purified. (A) *SCD1* mRNA expression was measured by RT-qPCR to establish the time of optimal knockdown. At 72 h after siRNA transfection *SCD1* mRNA was significantly reduced. (B) At 72 h after SCD1 siRNA transfection profibrotic genes along with *SCD1* were significantly downregulated and *PPARG* was upregulated. (C) Protein expression of αSMA and SCD1 quantification corresponds to Western blots in Fig. S5. Human GAPDH was used as internal control in both RT-qPCR and densitometric assessments. (D) Secreted Col1α1 in culture media from SCD1 siRNA transfected LX-2 was measured with ELISA using human pro-collagen 1a1 as standard. Significant reduction of Col1α1 secretion from culture media at 72 h after siRNA transfection indicate downregulation of Col1α1 protein in cells. Results are reported as means ± SEM (n = 3). ∗*p* <0.05, ∗∗*p* <0.01, ∗∗∗*p* <0.001 using 2-tailed Student's *t* test with a 95% confidence interval. HSC, hepatic stellate cell; ns, non-significant; PPARG, peroxisome proliferator-activated receptor gamma; SCD1, stearoyl CoA-desaturase 1.

To reinforce the inhibitory effect of SCD1 reduction on fibrogenesis, we silenced *SCD1* mRNA by transfecting human *SCD1* siRNA into LX-2 cells, and then measured *SCD1* mRNA and protein up to 96 h later. Transfection of non-targeting red fluorescent siRNA served as a negative control (Fig. S2). There was significant inhibition of *SCD1* mRNA expression at 72 h (68% *vs.* untransfected) quantified by RT-qPCR (Fig. 5A) whereas non-targeting red fluorescent siRNA had no effect on *SCD1* mRNA or protein expression (Fig. 5A and C). SCD1 protein expression was reduced in siRNA transfected cells at 72 h (Fig. 5C and S5).

We analysed mRNA expression for fibrogenic genes and proteins in LX-2 following *SCD1* knockdown. There was a time-dependent significant inhibition of *ACTA2*, *COL1A1*, *bPDGFR*, and *MMP2* mRNAs and upregulation of *PPARG* mRNA at 72 h post-transfection (Fig. 5B) similar to effects of Aramchol on LX-2 cells and phHSCs (Fig. 1A and B). LX-2 cells with *SCD1* knockdown also had reduced Col1α1 secretion at 72 h (Fig. 5D) mimicking the effect of inhibition using Aramchol (Fig. 2C). After 72 h of SCD1 knockdown αSMA and SCD1 protein expression by Western blot were reduced by 37% and 55%, respectively, compared with untransfected cells which persisted up to 96 h (Fig. 5C and S5). There was no significant difference in αSMA and SCD1 protein expression between untransfected cells and those transfected with a non-targeting siRNA (Fig. 5C and S5).

### Activity of Aramchol towards HSCs is attributable to SCD1 inhibition

**Fig. 6:**
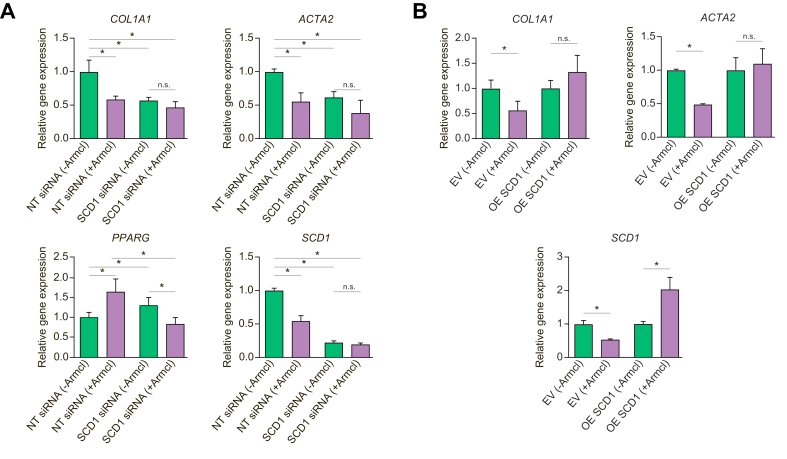
**Aramchol does not further reduce HSC activation in SCD1-knockdown HSCs whereas Aramchol does not suppress fibrogenesis if SCD1 is overexpressed**. (A) LX2 cells were transfected either with non-targeting siRNA or SCD1 siRNA for 24 h. After 24 h of transfection cells were maintained in either DMSO (vehicle) or 10 μM Aramchol for an additional 48 h. In the presence of SCD1 knockdown there was no additional downregulation of *COL1A1*, *ACTA2*, and *SCD1* or upregulation of *PPARG* by Aramchol. (B) LX-2 cells transiently transfected with *SCD1* or empty vector were incubated with or without 10 μM Aramchol for 48 h. *COL1A1*, *ACTA2*, and *SCD1* mRNA expression were assessed by RT-qPCR using *GAPDH* as internal control. Expression of *COL1A1* or *ACTA2* mRNAs was not downregulated by Aramchol treatment when SCD1 was overexpressed. Results are shown as mean ± SEM (n = 3). ∗*p* <0.05, ∗∗*p* <0.01, ∗∗∗*p* <0.001 using 2-tailed Student's *t* test with a 95% confidence interval. EV, empty vector; HSCs, hepatic stellate cells; ns, non-significant; NT siRNA, non-targeting siRNA; OE SCD1, overexpressed SCD1; PPARG, peroxisome proliferator-activated receptor gamma; SCD1, stearoyl CoA-desaturase 1.

To confirm that inhibitory effects of Aramchol are mediated by SCD1 inhibition in HSCs as reported previously,^27^ we examined whether the Aramchol further decreased fibrogenic gene expression if *SCD1* expression was first silenced using siRNA. As expected, LX-2 cells with *SCD1* siRNA knockdown had reduced gene expression of *SCD1*, *COL1A1*, *ACTA2*, and increased *PPARG* mRNAs, whereas addition of 10 μM Aramchol for 48 h elicited no additional inhibitory effect (Fig. 6A). Conversely, in LX-2 overexpressing SCD1, Aramchol no longer suppressed fibrogenic gene expression (Fig. 6B).

### Aramchol inhibits SCD1 and induces PPARG in primary human hepatocytes

**Fig. 7:**
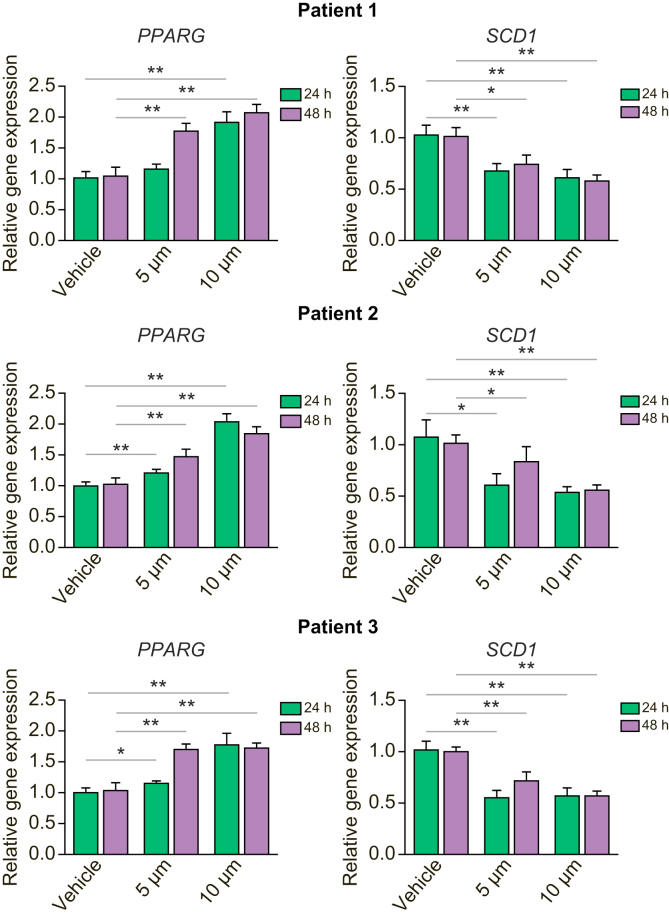
Aramchol downregulates *SCD1* and upregulates *PPARG* in primary human hepatocytes. Primary human hepatocytes isolated from 3 donors were treated with 5 μM and 10 μM Aramchol or DMSO (vehicle) for 24 or 48 h. Dose-dependent downregulation of *SCD1*, and upregulation of *PPARG* mRNA expression were quantified with RT-qPCR using *GAPDH* as an internal control. Results are reported as means ± SEM (n = 3). ∗*p* <0.05, ∗∗*p* <0.01, ∗∗∗*p* <0.001 using 2-tailed Student's *t* test with a 95% confidence interval. PPARG, peroxisome proliferator-activated receptor gamma; SCD1, stearoyl CoA-desaturase 1.

Activation of PPARγ signalling and inhibition of SCD1 in hepatocytes exert a beneficial effect on NASH.^28^^,^^29^ We confirmed that Aramchol displays these therapeutic activities in primary hepatocytes (Fig. 7). In 3 isolates of primary hepatocytes, Aramchol dose-dependently inhibited gene expression for *SCD1* while increasing *PPARG* (Fig. 7).

## Discussion

Aramchol is an orally active, liver-targeted, fatty acid-bile acid conjugate that is presently being evaluated in a phase III study for NASH and fibrosis. In a 1-year phase IIb study, Aramchol reduced liver fat, promoted NASH resolution, and improved liver biochemistries and fibrosis, with excellent safety and tolerability profiles.^12^ The mechanism by which Aramchol exerts its clinical activity is through downregulation of SCD1 in hepatocytes, but its impact on HSCs, the principal fibrogenic cell in liver, has not been explored. The mechanism underlying the inhibitory effect of Aramchol on SCD1 is unknown.

In this study, we demonstrate a direct antifibrotic activity of Aramchol on HSCs through downregulation of SCD1, as its inhibition either through drug treatment or by direct siRNA-mediated knockdown yielded parallel effects on HSC fibrogenesis. The effect on SCD1 is a component of a broader impact on both fibrogenic genes as well as mediators of cholesterol homeostasis that collectively attenuate HSC activation. The findings reinforce the pleiotropic effects of this drug, and further justify its evaluation as a novel therapy for NASH. Because NASH is a multifactorial disease there are many molecules currently under development that have multiple targets of action, including combination therapies that are emerging as an important principle of disease therapy. Of these many therapies, Aramchol is the only one that targets the downregulation of SCD1. Concurrent with this mechanism of action, the drug also induces PPARγ in both HSCs and hepatocytes, which is a pathway shown to benefit NASH pathology.^29^ As the pathogenesis of NASH reflects a convergence of multiple inputs with no clear hierarchy of causality to date, manipulating parallel pathways simultaneously through drugs that have multiple targets of action is an especially attractive strategy to prevent NASH development and progression.^30^

Interestingly, the antifibrotic effect of Aramchol is detectable in HSCs before changes in *SCD1* mRNA, suggesting that in addition to downregulation of *SCD1* there may be other mechanisms of the antifibrotic activity of the drug in this cell type. Indeed, the transcriptomic analysis in our study revealed the induction of *ABCA1* and *ABCG1* mRNAs, which encode cholesterol efflux transporters. As increased free cholesterol accumulation in HSCs stimulates their activation, enhanced cholesterol efflux could reduce intracellular cholesterol levels, thereby attenuating activation.^23^ Indirectly, these findings also support the reliance of HSC activation on autophagy to generate lipids for energy to support cellular activation.^4^ Therefore, enhanced flux of cholesterol could deprive cells of an important energy source required to fuel the many pathways associated with HSC activation.

Another interesting implication of these findings is the overlap in gene expression changes between effects of Aramchol and those inhibiting EMT, including hedgehog, which has been implicated in hepatic fibrosis through its effects on HSCs. EMT has largely been studied in neoplasia, but its contribution to organ fibrosis is unproven. Although EMT is not likely to be a major source of fibrogenic cells in hepatic fibrosis, the genetic similarities further reinforce the broad impact of Aramchol on HSC phenotype and function.

Although the findings support a potential direct antifibrotic effect of Aramchol in NASH patients, further investigation is needed to understand the additional mechanisms besides SCD1 inhibition by which Aramchol exerts its antifibrotic effects in patients. Nonetheless, the compelling safety and tolerability of this agent, combined with early evidence of *in vivo* efficacy, reinforce its potential utility for the treatment of NASH and fibrosis.

## Financial support

This work was supported by 10.13039/100000002NIH grants DK56621 (S.L.F.), R01CA172086, Plan Nacional of I+D SAF2017-88041-R, CIBERehd (J.M.M.) and a research contract from Galmed Pharmaceuticals. A.V. and S.L.F. are supported by the 10.13039/100000005Department of Defense (CA150272P3).

## Authors’ contributions

Study design: J.M.M., D.T., L.H., S.L.F. Performed genomics experiments: A.C., A.V. Performed cell culture experiments: D.B., B.B., F.L.O. Performed primary human hepatocyte (phHep) experiments and related data analyses: V.C. Performed cell culture experiments: D.F.R. Data interpretation: D.B., B.B., J.M.M., D.F.R., F.L.O., D.T., L.H., S.L.F. phHep data interpretation: R.E.S. Data interpretation and presentation: A.C., A.V. Manuscript preparation: D.B., B.B., S.L.F. Manuscript editing: J.M.M., A.C., D.T., L.H., R.E.S., A.V. Submission: S.L.F.

## Data availability statement

Raw RNA sequencing data have been deposited in ArrayExpress under accession number E-MTAB-9870 and are freely available.

## Conflicts of interest

D.T. and L.H. are employees of Galmed Pharmaceuticals. S.L.F. is a consultant to Galmed Pharmaceuticals and has equity in the company. S.L.F. is also a consultant to the following companies: 89 Bio, 3BV Bio, Axcella Health, Blade Therapeutics, Brii Biosciences, Bristol Myers Squibb, Can-Fite Biopharma, hemomAb, Coherus, Escient Therapeutics, Forbion, Genevant, Genfit, Glycotest, Glympse Bio, Heparegenix, Janssen Pharmaceutical, Metacrine, Morphic Therapeutics, North Sea Therapeutics, Novartis, Novo Nordisk, Ono Pharmaceuticals, Pfizer Pharmaceuticals, Scholar Rock, Second Genome, Surrozen, and Viking Therapeutics. A.V. has received consulting fees from Guidepoint, Fujifilm, Boehringer Ingelheim, FirstWord, and MHLife Sciences; advisory board fees from Exact Sciences, Nucleix, Gilead and NGM Pharmaceuticals; and research support from Eisai. The other authors declare no conflicts of interest that pertain to this work.

Please refer to the accompanying ICMJE disclosure forms for further details.
