# Supplementary material for: Aramchol downregulates stearoyl CoA-desaturase 1 in hepatic stellate cells to attenuate cellular fibrogenesis
Source: JHEP Rep. 2021 Jan 28;3(3):100237. doi: 10.1016/j.jhepr.2021.100237 (PMC8189934; doi:10.1016/j.jhepr.2021.100237)
Supplement: Multimedia component 1 [file mmc1.pdf]

Aramchol downregulates stearoyl CoA-desaturase 1 in hepatic stellate cells to attenuate cellular fibrogenesis

Dipankar Bhattacharya, Brittany Basta, Jose M. Mato, Amanda Craig,
David Fernández-Ramos, Fernando Lopitz-Otsoa, Darya Tsvirkun, Liat Hayardeny,
Vasuretha Chandar, Robert E. Schwartz, Augusto Villanueva, Scott L. Friedman

Table of contents

Fig. S1……………………………………………………………………………………2

Fig. S2……………………………………………………………………………………3

Fig. S3……………………………………………………………………………………4

Fig. S4……………………………………………………………………………………5

Fig. S5……………………………………………………………………………………7

Table S1…………………………………………………………………………………8

Table S2…………………………………………………………………………………9

Fig. S1.

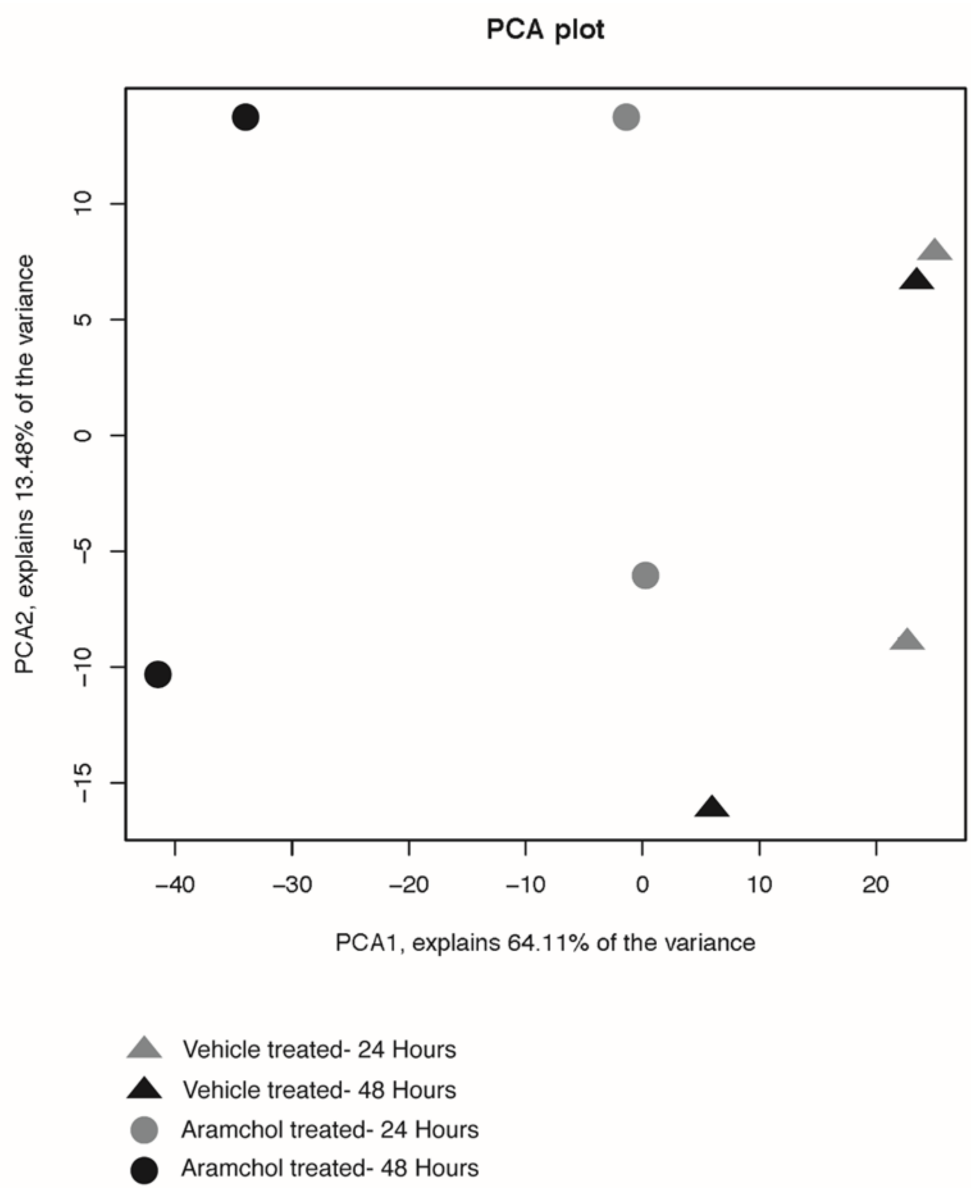

Fig. S1 Legend. Principle component analysis (PCA) of RNA-seq data. PCA1 is plotted against PCA2. Together, the first two PCAs captures 77.59% of the variance in the data. Distance between samples signifies differences in transcriptional profiles. Triangles represent vehicle treated samples and circles represent Aramchol treated samples. Black = treated-24 hours, Grey = treated-48 hours.

Fig. S2

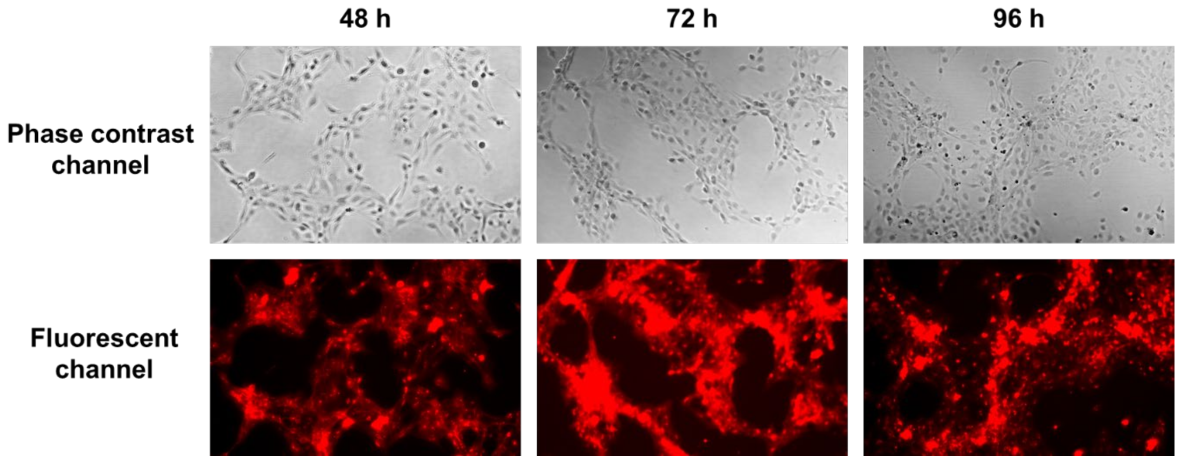

Fig. S2 Legend. siRNA transfection efficiency in HSCs. LX-2 cells were transfected with 0.5 micromolar nontargeting red fluorescent siRNA in presence of Accell siRNA delivery media without serum and antibiotics. The cells were cultured for 48-, 72- or 96 hours before harvested. Represented photomicrographs (10X magnification) of red fluorescence confirmed high transfection efficiency of siRNA in LX-2 cells

Fig. S3

A.

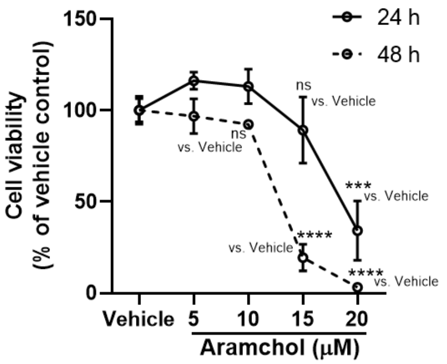

B.

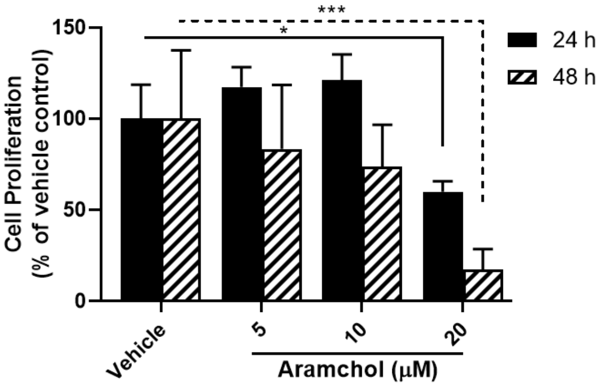

C.

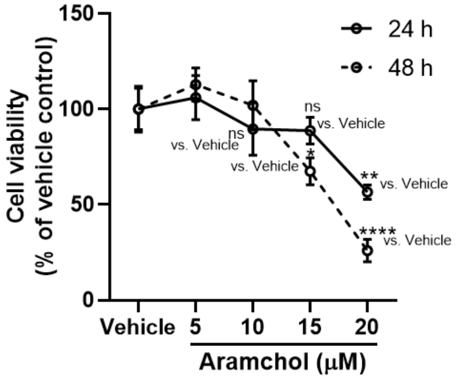

D.

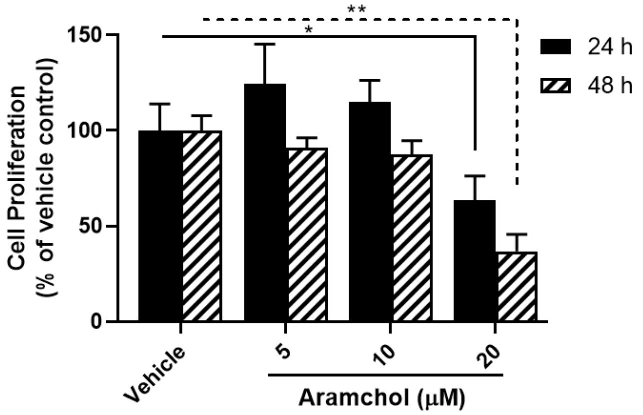

Fig. S3 Legend. Lack of toxicity of 10 μM Aramchol towards HSCs. Cytotoxicity and cell proliferation assays were performed in LX-2 cells (A and B) or primary human hepatic stellate cells (C and D) exposed up to 20 micromolar Aramchol for 48 hours. No toxic effect were found up to 10 micromolar drug treatment for 48 hours. No significant decrease of cell proliferation was detected at 10 micromolar Aramchol treatment for 48 hours. Results are reported as means ± SEM (n=3). *p<0.05, **p<0.01, ***p<0.001, ****p<0.001

Fig. S4

A.

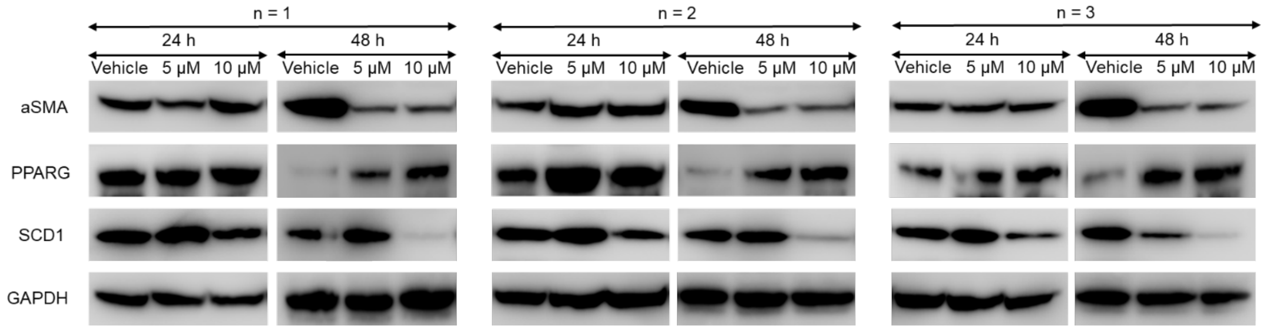

B.

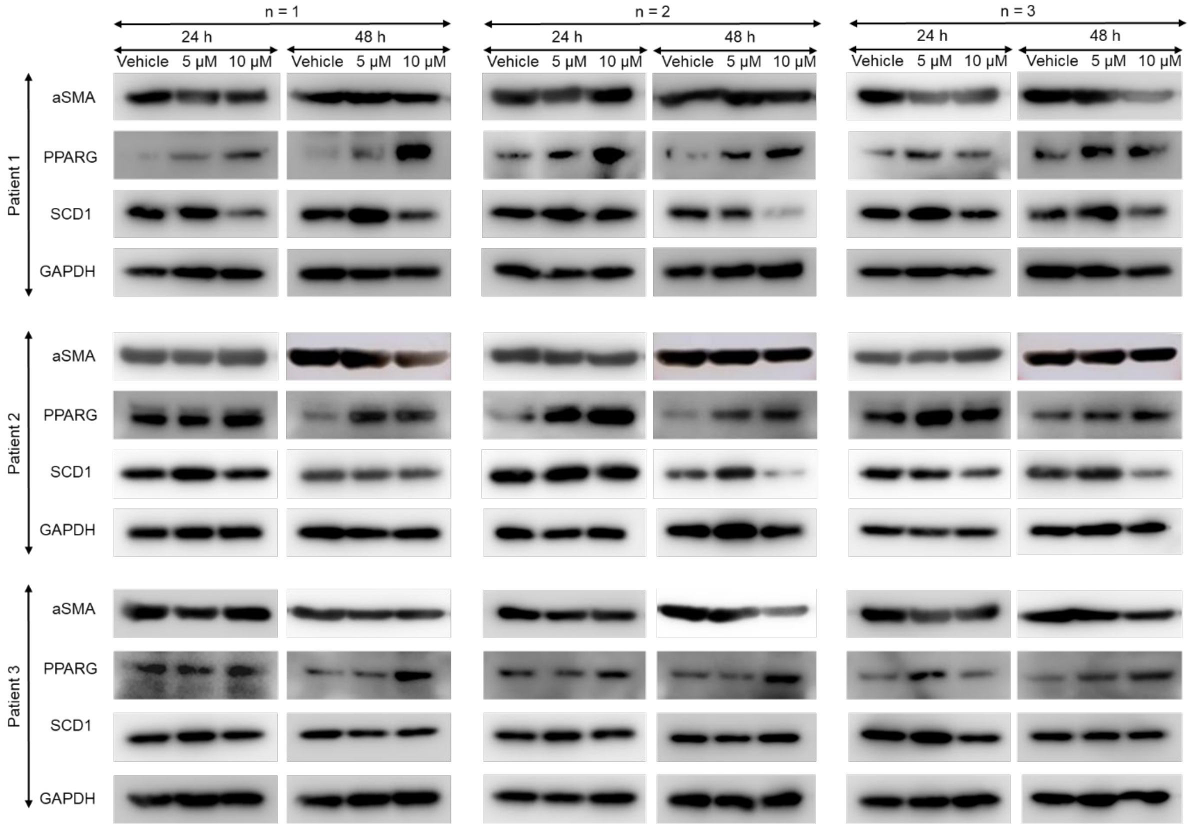

Fig. S4 Legend. Western blot images of HSC for quantification of protein expression. Western blot of LX-2 cells (A) and primary human hepatic stellate cells isolated from three (Patient 1, 2 &

3) donor livers (B) treated with 5 micromolar and 10 micromolar Aramchol or DMSO (as vehicle) for 24 or 48 hours. Western blot of alpha SMA, PPARG and SCD1 protein bands were imaged for quantification by densitometry shown in Figure 2A (LX-2) and 2B (primary human hepatic stellate cells). GAPDH expression was used as a control.

Fig. S5

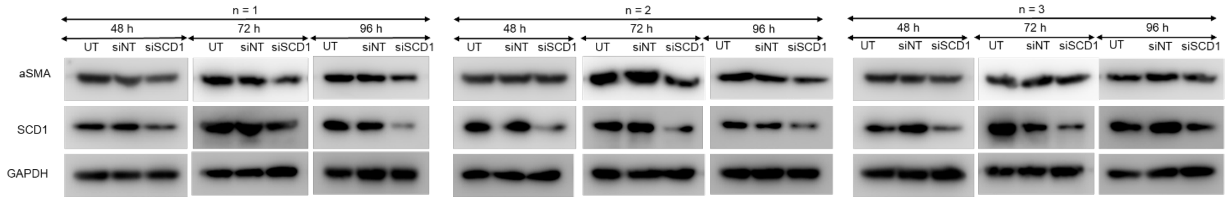

Fig. S5 . Western blot of siRNA transfected HSC for assessment of protein expression. LX-2 was transfected with either 1X siRNA buffer (untransfected) or non-targeting siRNA or pool of human *SCD1* siRNA for 48-, 72- and 96- hours. Western blot of alphaSMA and SCD1 protein bands were imaged for quantification by densitometry shown in Figure 5C. GAPDH expression was assessed for internal control. UT = Untransfected; siNT= Non-targeting siRNA; siSCD1 = SCD1 siRNA

Table S1. Human liver donors for primary hepatic stellate cell isolation

	Patient #	Sex	Age (Years)	Diagnosis	Fibrosis stage
**Primary human stellate cells**	1	Female	66	ICC NASH (Non-viral)	F0
	2	Male	49	Chronic hepatitis C infected	F3
	3	Male	65	Portal lymphoid inflammation with ductular reaction	F2

Table S2. Primer sequences for RT-qPCR

Gene	Sequences
ACTA2 Forward	5′ - AGGCACCCCTGAACCCCAA - 3′
ACTA2 Reverse	5′ - CAGCACCGCCTGGATAGCC - 3′
COL1A1 Forward	5′ - GGCTTCCCTGGTCTTCCTGG - 3′
COL1A1 Reverse	5′ - CCAGGGGGTCCAGCCAAT - 3′
bPDGFR Forward	5′ - CCAGAAGCCATCAGCAGCAAG - 3′
bPDGFR Reverse	5′ - AGGCCCTGAGAGATCTGTGG - 3′
MMP-2 Forward	5′ - CCCCAAGCTCATCGCAGAT - 3′
MMP-2 Reverse	5′ - GGTCCACGACGGCATCC - 3′
SCD-1 Forward	5′ - TGGAGGATTATCAGTATCACGATTTGC - 3′
SCD-1 Reverse	5′ - GTTTCCAGAATGAAGCCCAGAAGG - 3′
PPARG Forward	5′ - GACTTCTCCAGCATTTCTAC - 3′
PPARG Reverse	5′ - TCCACTTTGATTGCACTTTG - 3′
GAPDH Forward	5′ - CAATGACCCCTTCATTGACC - 3′
GAPDH Reverse	5′ - GATCTCGCTCCTGGAAGATG - 3′
