# Supplementary material for: Aramchol downregulates stearoyl CoA-desaturase 1 in hepatic stellate cells to attenuate cellular fibrogenesis
Source: JHEP Rep. 2021 Jan 28;3(3):100237. doi: 10.1016/j.jhepr.2021.100237 (PMC8189934; doi:10.1016/j.jhepr.2021.100237)
Supplement: Multimedia component 3 [file mmc3.pdf]

JHEP Reports
CTAT methods

Tables for a "Complete, Transparent, Accurate and Timely account" (CTAT) are now mandatory for all revised submissions. The aim is to enhance the reproducibility of methods.

- Only include the parts relevant to your study
- Refer to the CTAT in the main text as 'Supplementary CTAT Table'
- Do not add subheadings
- Add as many rows as needed to include all information
- Only include one item per row

If the CTAT form is not relevant to your study, please outline the reasons why:

1.1 Antibodies

Name	Citation	Supplier	Cat no.	Clone no.
Anti-alpha smooth muscle Actin antibody	Manufacturer website	Abcam	ab5694	Synthetic peptide corresponding to Human alpha smooth muscle Actin (N terminal)
Anti-PPAR gamma	Manufacturer website	Abcam	ab27649	Synthetic peptide conjugated to KLH derived from within residues 50 - 150 of Human PPAR gamma
Anti-SCD1 antibody	Manufacturer website	Abcam	ab19862	CD.E10
Anti-GAPDH Mouse mAb	PMID: 16005468	Millipore Sigma	CB1001	6C5
Peroxidase AffiniPure Goat Anti-Rabbit IgG, Fc fragment specific	Zhao, Q., et.al. (2019) Genome Med. 11, 23	Jackson Immuno Research Labs	111-035-008	RRID:AB_2337937
Anti-mouse IgG, HRP-linked Antibody	PMID: 32968434	Cell Signaling Technology	7076	N/A

1.2 Cell lines

Name	Citation	Supplier	Cat no.	Passage no.	Authentication test method
LX-2	PMID: 15591520	Developed in	N/A	18	1. Mycoplasma test

		Friedman Lab			2. Fibrogenic gene expression (RT-qPCR)<br>3. Profibrotic protein expression (western blot)<br><br>4. mRNA seq
Primary Human Hepatic Stellate Cell	PMID: 1735526	Cell isolation from 3 donors in Friedman lab	N/A	3	1. Mycoplasma test<br>2. Immunostaining: Desmin, GFAP, αSMA, CD31, CD68<br>3. Fibrogenic gene expression (RT-qPCR)<br>5. Profibrotic protein expression (western blot)<br>5. mRNA seq
Primary Human Hepatocytes	PMID: 26592180<br>PMID: 25092305	BioIVT (New York, NY)	00995-P	N/A	1. Secreted albumin and α1-Antitrypsin (AAT) by ELISA<br>2. CYP3A4 activity assay

1.3 Organisms – not applicable

Name	Citation	Supplier	Strain	Sex	Age	Overall n number

1.4 Sequence based reagents

Name	Sequence	Supplier
TruSeq® Stranded mRNA Library Prep	mRNA sequencing	Illumina

1.5 Biological samples – not applicable

Description	Source	Identifier

1.6 Deposited data

Name of repository	Identifier	Link
ArrayExpress	E-MTAB-9870	https://www.ebi.ac.uk/arrayexpress/

1.7 Software

Software name	Manufacturer	Version
Gen5 Microplate for Windows	BioTek Instruments	3.10
LightCycler 480 SW 1.5.1	Roche Diagnostics Corporation	1.5.1.62
2100 Expert	Agilent	B.02.11
STAR	GitHub	2.4.0c
RSeQC	SourceFroge	3.0.1
Picard	SourceForge	1.119
ImajeJ	NIH	1.52a
ZEN 2.6 (blue edition)	Carl Zeiss	2.6.76.00000
GraphPAD Prism		8.0.1

1.8 Other (*e.g.* drugs, proteins, vectors etc.)

**Drug** - Aramchol (arachidyl amido cholanoic acid) (F.W. 702.118)	**Manufacturer** - Galmed Pharmaceuticals	**Purity** - >99%
**siRNA** – SCD1 SMART pool (Accession no NM_005063); red fluorescent non-targeting control pool siRNA	**Manufacturer** - Horizon Discovery	**Cat. no:** E-005061-00-0010 (for SCD1); D-001910-10-20 (for non-targeting control)
**Vector** - pCMV6-Entry plasmid ; pCMV6-SCD1 plasmid (SCD1 Accession no NM_005063)	**Manufacturer** - Origene	**Cat. no:** PS100001 (for Entry plasmid) ; RC209148 (for human SCD1 plasmid)

1.9 Please provide the details of the corresponding methods author for the manuscript:

Dipankar Bhattacharya, MS
Box 1123, Icahn School of Medicine at Mount Sinai
1425 Madison Ave, Room 1176
New York, NY 10025, USA
Tel (212) 659-9524
Fax 212 849 2574
Email: Dipankar.bhattacharya@mssm.edu

2.0 Please confirm for randomised controlled trials all versions of the clinical protocol are included in the submission. These will be published online as supplementary information.

**Not applicable**
